# Neuropsychological Performance Is Correlated With Tau Protein Deposition and Glucose Metabolism in Patients With Alzheimer’s Disease

**DOI:** 10.3389/fnagi.2022.841942

**Published:** 2022-05-18

**Authors:** Zhen Qiao, Guihong Wang, Xiaobin Zhao, Kai Wang, Di Fan, Qian Chen, Lin Ai

**Affiliations:** ^1^Department of Nuclear Medicine, Beijing Tiantan Hospital, Capital Medical University, Beijing, China; ^2^Department of Neurology, Beijing Tiantan Hospital, Capital Medical University, Beijing, China

**Keywords:** Alzheimer’s disease, glucose metabolism, tau protein, positron emission tomography, neuropsychological scores, neuropsychological domain

## Abstract

**Objective:**

This study characterizes glucose metabolism and tau protein deposition distribution in patients with Alzheimer’s disease (AD) and to evaluate the relationships between neuropsychological performance and tau protein deposition or glucose metabolism using ^18^F-FDG and ^18^F-AV1451 positron emission tomography/computed tomography (PET/CT).

**Methods:**

Sixty-four patients with β-amyloid-positive (Aβ+) AD and twenty-five healthy participants were enrolled in this study. All participants underwent ^18^F-FDG and ^18^F-AV1451 PET/CT. Clinical data and neuropsychological scores were collected. Patients with AD were divided into mild, moderate, and severe groups according to mini-mental state examination (MMSE) scores. The standardized uptake value ratios (SUVRs) for both FDG and AV1451 PET images were calculated using the cerebellar vermis as reference. The SUVRs of the whole cerebral cortex and each brain region were calculated. The volume of interest (VOI) was obtained using automated anatomical atlas (AAL) and Brodmann regions. Student’s *t*-test was used to perform intergroup comparisons of SUVR. The partial correlation coefficient between SUVR and neuropsychological scores was computed in an inter-subject manner using age and education as covariates.

**Results:**

The mild subgroup showed a reduction in glucose metabolism and aggregation of tau protein in the temporoparietal cortex. With a decline in neuropsychiatric performance, the SUVR on FDG PET decreased and SUVR on tau PET increased gradually. The areas of glucose metabolism reduction and tau protein deposition appeared first in the parietal cortex, followed by the temporal and frontal cortex, successively. Both FDG and tau SUVRs significantly correlated with MMSE, Montreal cognitive assessment (MOCA), auditory verbal learning test (AVLT), Boston naming test (BNT), clock drawing task (CDT), and verbal fluency test (VFT) (*p* < 0.05). The SUVR on FDG PET significantly correlated with activities of daily living (ADL) and the Hamilton depression scale (HAMD). There was no significant correlation between the tau SUVRs and ADL or HAMD.

**Conclusion:**

The extension of tau protein deposition was similar but not exactly consistent with the area of glucose metabolism reduction. Both tau and FDG SUVRs correlated with cognitive function in domain-specific patterns, and the results of FDG PET more closely correlated with neuropsychological function than tau PET results did.

## Introduction

Alzheimer’s disease (AD) is a primary neurodegenerative disease and one of the most common clinical types of dementia, accounting for up to 70% of cases (Plassman et al., 2007). Clinical manifestations of AD mainly include memory loss and cognitive decline as well as behavioral dysfunction (Scheltens et al., 2016). The neuropathological hallmarks are the combined presence of senile plaques formed by the deposition of β-amyloid (Aβ) and neurofibrillary tangles (NFT) composed of over-phosphorylated tubule-associated tau proteins. Although Aβ plaques may play a key role in AD pathogenesis, histopathological study suggests that the level of Aβ deposition is a poor predictor of severity of cognitive impairment (Giannakopoulos et al., 2003). In contrast, the severity of cognitive impairment correlates best with the burden of neocortical NFTs (Nelson et al., 2012). Braak tangle staging is strongly associated with cognitive impairment (Sabbagh et al., 2010). Braak defined six stages in the evolution of NFT involvement of the dementing brain (Braak and Braak, 1991). Cognitive impairment occurred when the NFT expanded to the neocortex. Some studies on autopsy and cerebrospinal fluid examination (Nichols and Holmes, 2002; van Rossum et al., 2012; Kantarci et al., 2017) suggested that the relationship between tau protein and cognition was more obvious than that between Aβ and cognition. The advents of molecular imaging agents that provide quantitative measures of Aβ and tau have allowed researchers to explore these proteins in AD *in vivo*. Moreover, positron emission tomography (PET) imaging could be used to detect the spatial distribution of Aβ/tau protein and glucose metabolism *in vivo*. This provides the possibility to assess the spatial association of Aβ/tau protein and glucose metabolism. Amyloid PET is inadequate for assessing disease severity in patients with AD, as well as cognitive function in general (Rowe et al., 2007; Newberg et al., 2012; Smailagic et al., 2015). Flortaucipir (AV1451) has high affinity and selectivity for paired helical filament (PHF)-tau. Previous studies have shown that flortaucipir retention patterns mirror the Braak stages (Cho et al., 2016; Johnson et al., 2016; Smith et al., 2018; Lowe et al., 2019; Vogel et al., 2019), further validating flortaucipir PET as an *in vivo* marker for hyperphosphorylated tau. The amount and distribution of tau PET (AV1451) were shown to correlate with neuropsychological performance in some studies (Ossenkoppele et al., 2016). The distribution of tau protein in the brain was reported to be similar to low-metabolic regions on FDG PET imaging (Ossenkoppele et al., 2015, 2016; Bischof et al., 2016; Dronse et al., 2017). Glucose metabolism is the dominant source of energy in the brain and is closely associated with neuronal activity. A decline in glucose metabolism is an indicator of brain dysfunction or neuronal loss. Previous studies have shown that glucose metabolism was related to the severity of cognitive impairment (Chételat et al., 2005; Kuczynski et al., 2008). FDG PET quantification is a superior indicator of cognitive performance in patients with AD or mild cognitive impairment (MCI) over amyloid PET (Khosravi et al., 2019). It has recently been demonstrated that the aggregation of tau and the decline in glucose metabolism within functional cortical systems may underlie cognitive loss in different domains. However, the association between PET and neuropsychological performance across different domains remains to be fully characterized. Mapping the distribution of tau pathology and glucose metabolism in patients with varying levels of cognitive impairment may be helpful for our understanding of the disease mechanisms of AD. In this study, we grouped patients with AD according to their respective neuropsychological scores to assess the spatial distribution characteristics of glucose metabolism and tau protein deposition in each group of patients using FDG and tau PET. Correlation analysis between PET and neuropsychological scores was conducted. Our aim was to explore the link between different neuropsychological domains and FDG/tau PET and to explore the applications of tau and FDG PET in evaluating neuropsychological performance in AD. This study may help exploring the role of tau protein deposition in the pathogenesis of AD. Moreover, this study is testing the hypothesis that both tau and FDG PET can indicate a decline in neuropsychological domains in patients with AD.

## Materials and Methods

### Inclusion of Participants

This study was a retrospective analysis. Participants comprised patients who underwent ^18^F-FDG and ^18^F-AV1451 PET in the Department of Nuclear Medicine in Beijing Tiantan Hospital between June 2016 and April 2018. The interval between the scans did not exceed 2 weeks. All patients had visited the Department of Neurology due to cognitive decline. Patients with a clinical diagnosis of AD according to the NIA-AA (2011) and brain Aβ+ were included in this study. Aβ status was assessed using an ^18^F-AV45 or ^11^C-PIB PET scan. Physical and neuropsychiatric data of patients were collected. Neuropsychiatric assessments included mini-mental state examination (MMSE), Montreal cognitive assessment (MOCA), clock drawing task (CDT), auditory verbal learning test (AVLT), Boston naming test (BNT), verbal fluency test (VFT), Hamilton depression scale (HAMD), and activities of daily living (ADL). The AVLT score was the sum of the immediate and delayed recall. According to the MMSE score, patients with AD were divided into three groups, namely, mild (≥20), medium (10–20), and severe (≤10). The cognitively normal population, who were composed of the patients’ family members or those who visited PET centers during the study period, were enrolled as healthy control (HC) group if the MMSE score was ≥27, and ^18^F-AV45/^11^C-PIB PET/CT was negative. Both ^18^F-AV45 and ^11^C-PIB were amyloid PET radiotracers. Increased tracer uptake gray matter was assessed visually and defined as Aβ+: a loss of gray-white distinction, greater accumulation in the cortex than in the cerebellar gray matter, and in at least two AD specific cortical regions above the white matter or in at least one cortical region at the level of the white matter. Informed consent was obtained from all participants, and this study was approved by the Ethics Committee of Beijing Tiantan Hospital.

In addition, to evaluate the imaging characteristics of patients with different neuropsychiatric performances, patients with AD were grouped according to each neuropsychiatric domain: MOCA (mild, moderate, and severe), CDT (mild, moderate, and severe), AVLT (mild, moderate, and severe), BNT (mild, moderate, and severe), VFT (mild, moderate, and severe), HAMD (mild, moderate, and severe), and ADL (mild, moderate, and severe).

### Data Acquisition

All patients were required to fast for 4–6 h before the ^18^F-FDG PET scan, and the blood glucose level was maintained below 6.1 mmol/L. ^18^F-FDG was injected intravenously at 5.55 MBq/kg of body weight in a dim and quiet room. After 50 min, PET and low-dose CT data were acquired using a PET/CT scanner (Discovery Elite, GE, United States). The PET data were acquired in 3-dimensional acquisition mode, and low-dose CT data were used for attenuation correction of PET data. The acquired PET data were reconstructed using an Ordered Subjects Extension (OSEM) algorithm, with 18 subsets and 192 × 192 matrix. No special preparation was needed for patients before the ^18^F-AV1451 PET scan. The tracer was injected intravenously at 370–555 MBq. The image acquisition and reconstruction were the same as ^18^F-FDG PET.

### Positron Emission Tomography Data Preprocessing

The ^18^F-FDG and ^18^F-AV1451 PET images of all participants were preprocessed using Statistical Parametric Mapping (SPM8, Institute of Neurology, University College London, United Kingdom) software in MATLAB, version 2013b (MathWorks, United States).

First, all PET images were spatially normalized to Montreal Neurological Institute (MNI) space. ^18^F-AV1451 and ^18^F-FDG PET images were co-registered for each participant, and then, the ^18^F-FDG image was spatially normalized to MNI space *via* the FDG PET standard template present in SPM. Later, the parameters obtained from ^18^F-FDG image normalization were applied to the co-registered ^18^F-AV1451 PET image, resulting in normalized tau-PET images in MNI space. The spatially normalized ^18^F-FDG and ^18^F-AV1451 PET images were then smoothed using a Gaussian kernel with an 8-mm full width at half maximum (FWHM).

### Statistical Analysis

The participants’ characteristics were summarized using descriptive statistics. Continuous variables with a normal distribution are presented as mean ± SD.

The standardized uptake value ratios (SUVRs) for both FDG and AV1451 PET images were calculated using the cerebellar vermis as a reference. In this study, SUVRs in the whole cerebral cortex and each brain region were calculated. The brain regions selected for this study included the frontal, parietal, temporal, and occipital lobes, hippocampus, cuneus, precuneus, and cingulum (anterior/posterior). The volume of interest (VOI) mentioned above was obtained using automated anatomical atlas (AAL) and Brodmann regions. Student’s *t*-test was used to perform intergroup comparisons of SUVR values using SPSS, and *p* < 0.05 was considered significant. To assess the spread of tau protein deposition and reduction of glucose metabolism reduction, Student’s *t*-test was used to perform intergroup comparisons of SUVR images using SPM with significant differences. The percentage of each lobe that was affected was calculated by the number of voxels in each lobe divided by the total number of voxels with a significant difference to assess the distribution of tau protein deposition and glucose metabolism reduction.

To clarify the association between cognition and tau protein deposition and glucose metabolism, we conducted a related analysis. The partial correlation coefficient between the SUVR values of the whole cerebral cortex/VOI and neuropsychiatric scores was computed in an inter-subject manner using SPSS. In the correlation analysis, age and education were considered as covariates, and *p* < 0.05 was considered statistically significant. For intuitively observing the distribution of correlation coefficients, partial correlation analyses for comparing voxel-wise SUVR images and neuropsychiatric scores were conducted with age and education as covariates using RESTPlus version 1.24. R images were formed, composed of unique correlation coefficients for each voxel in the brain between the SUVR and cognitive scores. The mapping image for *R* > 0.4 was conducted using xjView.

## Results

### Participant Sample

The clinical data of 64 patients with AD and twenty-five HCs in this study are provided in Tables 1, 2. Table 1 shows the demographic data of the MMSE subgroup. Table 2 shows the neuropsychiatric scores of different subgroups.

**TABLE 1:** Clinical data of Alzheimer’s disease (AD) and healthy control (HC) groups.

	Mild AD (*n* = 17)	Moderate AD (*n* = 34)	Severe AD (*n* = 13)	HC group (*n* = 25)
Age (years; mean ± SD)	64.00 ± 7.11	64.79 ± 8.64	60.85 ± 6.91	61.96 ± 10.94
Male/female	8/9	13/21	5/8	17/8
Education level (years; mean ± SD)	11.06 ± 3.80	10.32 ± 4.04	7.85 ± 4.32	11.83 ± 4.04
Course of disease (years; mean ± SD)	2.59 ± 1.36	3.09 ± 1.82	2.69 ± 1.44	
MMSE (mean ± SD)	24.00 ± 2.12	15.68 ± 2.77	4.31 ± 2.92	27.96 ± 1.31

**TABLE 2:** Neuropsychiatric scores of different subgroups.

Scores	Mild AD group	Moderate AD group	Severe AD group
MOCA (mean ± SD)	18.18 ± 2.40	11.65 ± 1.32	4.72 ± 2.14
AVLT (mean ± SD)	28.35 ± 3.78	17.76 ± 2.70	4.73 ± 5.21
BNT (range)	7–22	4–21	1–9
VFT (mean ± SD)	14.93 ± 2.58	9.50 ± 1.25	4.20 ± 2.14
CDT (range)	1–5	0–5	0–4
ADL (range)	8–11	8–21	9–23
HAMD (range)	0–21	0–15	0–15

### Intergroup Comparison of Positron Emission Tomography/Computed Tomography Imaging

The FDG PET image showed that the SUVR of the whole cerebral cortex in the AD group (0.98 ± 0.12) was significantly lower than that in the HC group (1.10 ± 0.10; *p* < 0.001). The tau PET image showed that the SUVR (1.35 ± 0.23) in the AD group was significantly higher than that in the HC group (1.10 ± 0.16; *p* < 0.001).

**FIGURE 1:**
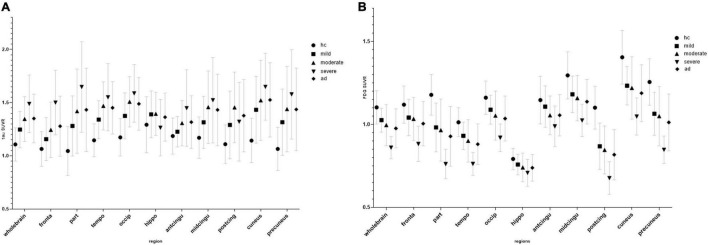
Regional SUVR of FDG **(A)** and tau positron emission tomography (PET) **(B)** images in the control group and mini-mental state examination (MMSE) subgroup.

**FIGURE 2:**
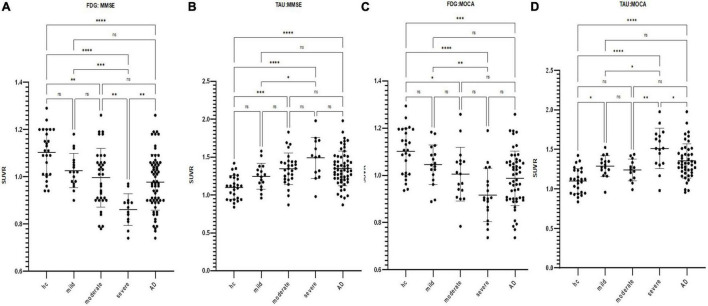
Scatterplot of the standardized uptake value ratio (SUVR) distribution in the cerebral cortex for FDG and tau PET images by subgroup according to respective neuropsychiatric scores (*p < 0.05, **p < 0.01, ***p < 0.001, ****p < 0.0001). **(A)** FDG SUVR distribution in the HC group and mild/moderate/severe AD subgroups as defined by MMSE. **(B)** Tau SUVR distribution in the HC group and mild/moderate/severe AD subgroups as defined by MMSE. **(C)** FDG SUVR distribution in the HC group and mild/moderate/severe AD subgroups as defined by MOCA. **(D)** Tau SUVR distribution in the HC group and mild/moderate/severe AD subgroups as defined by MOCA.

According to the MMSE score, the SUVR of the cerebral cortex on FDG PET in the mild, moderate, and severe AD groups was 1.03 ± 0.07, 1.0 ± 0.12, and 0.86 ± 0.07, respectively. The SUVR on tau PET in the mild, moderate, and severe AD groups were 1.25 ± 0.17, 1.35 ± 0.21, and 1.49 ± 0.27, respectively. The SUVR of each brain region is shown in Figure 1. Figure 2 shows the SUVR in different subgroups according to MMSE and MOCA scores. The SUVR on FDG PET decreased as all neuropsychiatric scores declined. The SUVR on tau PET increased gradually with declining MMSE, AVLT, VFT, BNT, and ADL.

**FIGURE 3:**
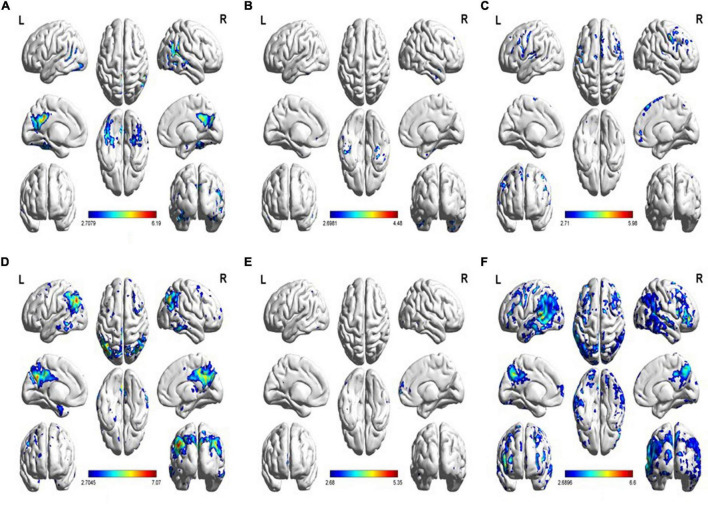
**(A)** Increased tau uptake in the MMSE-mild subgroup relative to the HC group. **(B)** Increased tau uptake in the MMSE-moderate subgroup relative to the MMSE-severe subgroup. **(C)** Increased tau uptake in the MMSE-severe subgroup relative to the MMSE-moderate subgroup. **(D)** Decreased FDG uptake in the MMSE-mild subgroup relative to the HC group. **(E)** Decreased FDG uptake in the MMSE-moderate subgroup relative to the MMSE-severe subgroup. **(F)** Decreased FDG uptake in the MMSE-severe subgroup relative to the MMSE-moderate subgroup (*p* < 0.01).

In the voxel-wise analysis, compared with the HC groups, glucose metabolism reduction in the mild AD group was located in the parietal lobe (48.5%) and temporal lobe (16.9%), and the right/left (R/L) was 1.03/1. The tau protein deposition area included the temporal lobe (39.2%), parietal lobe (22.7%), and occipital lobe (21.7%), and the R/L was 1.04/1. There was no significant difference between the moderate and mild groups (*p* < 0.001). Compared with the moderate groups, the areas with glucose metabolism reduction in the severe AD group were the frontal lobe (35.9%), temporal lobe (25.6%), and parietal lobe (23.2%), and the R/L was 1.82/1. The distribution of tau protein deposition in the severe AD group was the frontal lobe (96.6%), and the R/L was 1/1. The result is shown in Figure 3. The mild subgroup in all neuropsychiatric domains showed a reduction in glucose metabolism and aggregation of tau protein in the temporoparietal cortex. As neuropsychiatric scores declined, glucose metabolism reduction and tau protein deposition successively affected the parietal, temporal, and frontal lobes.

### Correlation Between Cognitive Scores and Positron Emission Tomography

**FIGURE 4:**
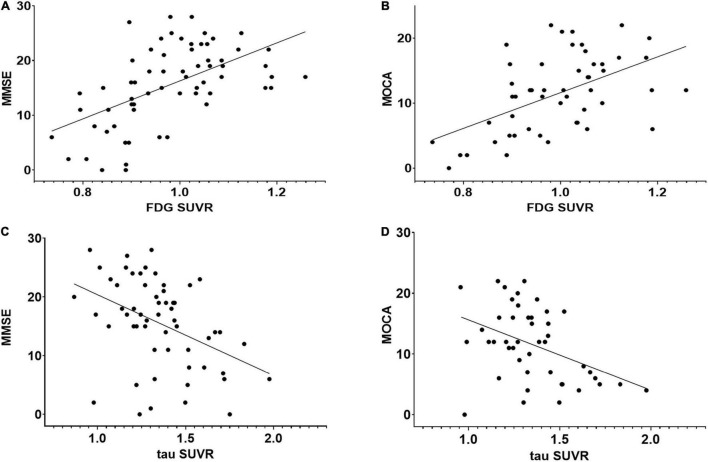
Regression plots representing the relationship between neuropsychological scores and FDG or tau PET SUVR in the whole cortex. **(A)** Relationship between MMSE and FDG SUVR (*r* = 0.557, *p* < 0.01). **(B)** Relationship between MOCA and FDG SUVR (*r* = 0.501, *p* < 0.01). **(C)** Relationship between MMSE and tau SUVR (*r* = –0.411, *p* < 0.01). **(D)** Relationship between MOCA and tau SUVR (*r* = –0.402, *p* < 0.01). There was no significant correlation between tau PET and FDG PET SUVRs.

**FIGURE 5:**
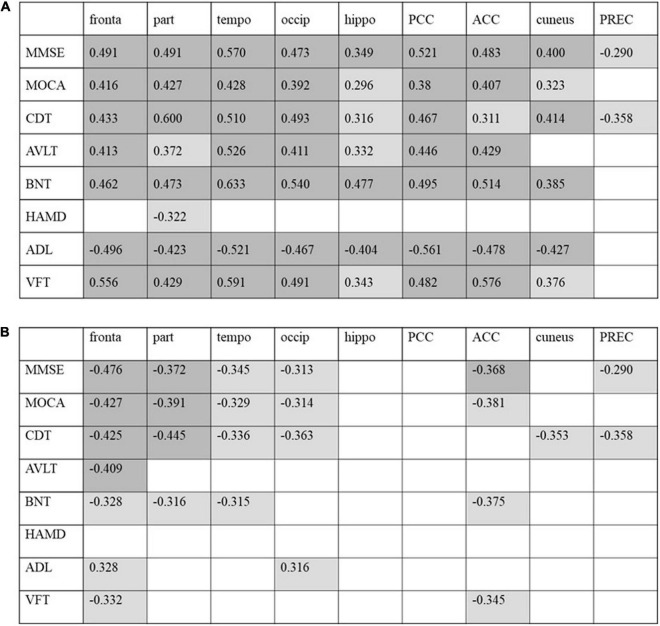
Correlation between cognitive scores with regional FDG-PET SUVR **(A)** and tau-PET SUVR **(B)** in patients with AD. Correlation coefficients with *p* < 0.05 are shaded in light gray, and results with *p* < 0.01 are shaded in dark gray. PCC, posterior cingulate cortex; ACC, anterior cingulate cortex; PREC, precuneus.

**FIGURE 6:**
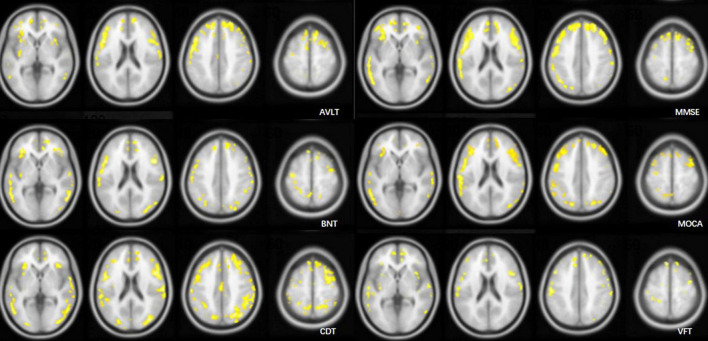
Cluster maps overlaid on an MRI T1WI atlas. Clusters included >100 voxels with a correlation coefficient (*R*) > 0.4 in voxel-wise relationship analysis of the AV1451 distribution and cognitive scores.

Both whole-brain cortical SUVRs of FDG and tau PET significantly correlated with MMSE, MOCA, AVLT, BNT, CDT, and VFT (*p* < 0.001; Figure 4). The SUVRs on FDG PET significantly correlated with ADL and HAMD. There was no significant correlation between SUVRs on tau PET and ADL or HAMD. Several structural regions were analyzed with the corresponding neuropsychiatric score, and the results are shown in Figure 5. The correlation coefficient distribution maps of the relationship between tau SUVR and cognitive scores are shown in Figure 6.

## Discussion

Previous studies have shown that Aβ protein deposition can also be seen in healthy populations (Hebert et al., 1995; Ganguli et al., 2000; Kukull et al., 2002; Sperling et al., 2011), and all diseases featuring abnormal Aβ deposition were included in the Alzheimer’s continuum in the framework of AD published by NIA-AA in 2018 (Jack et al., 2018) for observational and interventional studies. Aβ protein deposition was used as a screening criterion for patients in this study, and the HCs in this study did not have Aβ protein deposition (Chételat et al., 2020).

The FDG SUVR reduced and tau SUVR increased with neuropsychiatric functional decline in most domains. The exception is that the SUVR on tau PET in the MOCA/CDT-moderate subgroup was lower compared with the respective mild subgroup. The extent of glucose metabolism decrease was more significant than that of tau protein deposition in patients in the severe subgroup (MMSE, VFT, BNT, and AVLT) compared with the moderate subgroup. VOI and voxel-wise statistical analysis of the whole brain was conducted. Voxel-wise statistical analysis of the whole brain revealed that the tau protein deposition area in the mild subgroup (MMSE and MOCA) was mainly distributed in the temporal lobe, which also included the entorhinal cortex and hippocampus (Braak I-II) and amygdala nucleus (Braak III-IV) followed by the parietal lobe. As the disease progresses, the frontal lobe is involved. According to the Braak staging system (Braak and Braak, 1991, 1995; Braak et al., 2011), NFTs successively affected the entorhinal cortex and hippocampus, limbic system, and neocortex. Braak stages correlate with clinical symptoms and severity of disease, and Braak I-II is characterized by a lack of significant symptoms of cognitive decline. When the neocortex is involved, patients can show a decrease in cognitive function, social function, and executive function. A similar change pattern was shown on FDG PET imaging. The only difference is that the area with glucose metabolism decreases in the mild subgroup was mainly located in the parietal lobe, and tau protein deposition was in the temporal lobe. Nevertheless, tau PET imaging is not only a mirror image of glucose metabolism. Tau protein deposition was suggested as a facilitator of the downstream of Aβ (Jack et al., 2010, 2013; Jack and Holtzman, 2013). Tau protein deposition has a devastating effect on synaptic function and may be an initiator of cognitive decline. A reduction in glucose metabolism may be a consequence of tau protein deposition. Glucose metabolism of the parietal lobe may be decreased before the tau protein spread to the parietal lobe. With the progress of the disease, the spread rate of glucose metabolism decrease is faster than that of tau protein deposition. A previous study (Iaccarino et al., 2021) showed that the spatial extent of tau accumulation typically exceeded and contained the area of neurodegeneration (FDG PET), and a small number of participants showed that the neurodegeneration extent exceeded that of tau accumulation. Age and comorbidities are considered factors that contribute to neurodegeneration. Moreover, the distribution of glucose metabolism reduction and tau protein deposition is inconsistent (Brier et al., 2016; Sepulcre et al., 2017; Aschenbrenner et al., 2018). Tau protein deposition may affect the stability of neighboring neuronal cells and even affect brain network connectivity. Tau protein deposition is not the only factor in reducing glucose metabolism. Whether both Aβ and tau are mechanistically required to trigger a reduction in glucose metabolism is not yet fully understood. Some scholars suggest that there are other factors (gene mutation and infection) (Tiraboschi et al., 2004; van der Flier et al., 2011; Ossenkoppele et al., 2013; Lehmann et al., 2014) that induce neurodegeneration, while the deposition of amyloid and tau proteins is the only accompanying pathological change. Comparison at the individual level may be necessary to compare the extent of tau protein deposition and glucose metabolism and explore the association between tau protein deposition and glucose metabolism.

Voxel-wise comparison results showed that the tau protein deposition pattern in MMSE subgroups was similar to that in MOCA subgroups. Both MMSE and MOCA are routine cognitive screening tests that cover various cognitive domains. Tau protein deposition extended to the temporoparietal cortex in the mild subgroups of multiple cognitive domains. The similarity in the results may be because the participants included in this study were duplicated. There were cognitive declines in multiple domains in each participant.

Correlation analysis was conducted to assess the relationship between SUVR and neuropsychiatric performance. Tau SUVR negatively correlated with cognitive scores. The severity of AD symptoms correlates well with increasing tau deposition. Koychev et al. (2017) performed a longitudinal analysis to compare cognitive assessments and CSF or PET imaging measurements of Aβ and tau at baseline and 6 months in patients with mild AD and found that the tau protein measured on PET and in the CSF of the elderly patient group related to cognitive decline at 6 months, although the decline in cognitive ability at 6 months was not significant. An association between the increasing SUVR on tau PET and decline in cognitive scores was observed within the neocortex (frontal/temporal/parietal/occipital lobe) as shown in Figure 5. Gomperts et al. (2016) analyzed the regions of interest in patients with dementia with Lewy bodies and Parkinson’s disease and found a strong correlation between Clinical Dementia Rating (CDR) and MMSE scores and tau protein deposition in the bilateral inferior temporal lobes and precuneus. There was no correlation between cognitive scores and the SUVR on tau PET in the hippocampus, which was the earliest area with tau protein deposition in this study. The SUVR on tau PET in the cuneus and precuneus did not correlate with cognitive scores. Zhao et al. (2019) observed a decline in MMSE score with an increase in AV1451 binding in the amygdala, entorhinal cortex, parahippocampus, and fusiform. This study is inconsistent with the above findings due to population and methodology differences. The areas affected by tau protein deposition may have less cognitive function decline. Baghel et al.’s (2019) study showed a negative association between tau deposition regional retention (precuneus) and MMSE score, and a positive association between regional FDG uptake (precuneus) and MMSE score. We also observed a significant relationship between FDG SUVR in the neocortex and cognitive scores as shown in Figure 5. The extension was larger than that on tau PET. FDG uptake in the hippocampus, cingulate gyrus, cuneus, and precuneus correlated with the MMSE/MOCA scores. This may be because FDG uptake in these regions is associated with cortical FDG uptake which may affect cognitive function.

The FDG uptake was related to HAMD. Patients with depression had reduced glucose metabolism in the cerebral cortex and a negative correlation between glucose metabolism in the frontal lobe and HAMD score (Su et al., 2014). However, no significant correlation between HAMD and tau SUVR was shown. Depression in patients with AD may be due to the combination of impairment of daily living and workability and the social and family environment and may have less to do with tau protein deposition.

Analysis of spatial distribution patterns may be helpful for researching brain function. The MMSE (Ossenkoppele et al., 2016) score was suggested to negatively correlate with tau SUVR in the bilateral orbital frontal cortex and the left anterior temporal cortex in patients with AD. It is suggested that the loss of visual space, language memory, and other functions are related to tau PET (Devous et al., 2021). The voxel-wise correlation pattern analysis between neuropsychiatric scores and SUVR was not unique to our study (Devous et al., 2021). Voxel-based analysis of tau PET showed that most of the area correlating with memory function (AVLT) was located in the frontal cortex, temporal cortex, and insula. Language skills are assessed with measures of fluency, naming, and responding to instructions. The area correlating with VFT focuses on assessing fluency distributed in the frontal cortex. The region correlating with BNT is distributed in the parietotemporal cortex and frontal cortex. Spatial function (CDT) correlated with tau protein distribution across extensive neocortical areas, with a significant correlation in the left parietal cortex. The frontal lobe is involved in almost all neuropsychiatric functions, including memory, language, and intelligence. The correlation coefficient of FDG PET with cognitive scores is higher than that of tau PET with no statistical difference in most regions. FDG PET may be more effective in reflecting neuropsychiatric performance than tau PET.

The relationship between tau protein deposition and glucose metabolism reduction was less evident in this study. Further work incorporating longitudinal studies is necessary to evaluate the correlations between neuropsychiatric performance and tau protein deposition and understand the role of tau protein deposition in the pathogenesis of AD.

## Data Availability Statement

The original contributions presented in the study are included in the article/supplementary material, further inquiries can be directed to the corresponding authors.

## Ethics Statement

The studies involving human participants were reviewed and approved by the Ethics Committee of Beijing Tiantan Hospital. The patients/participants provided their written informed consent to participate in this study.

## Author Contributions

ZQ analyzed the image data and wrote the manuscript. LA supervised the project. GW contributed to patient inclusion and clinical data collection. XZ and KW performed the data acquisition and analysis. DF synthesized the tracer. All authors contributed to the conception and design of the study, commented on the previous versions of the manuscript, and read and approved the final manuscript.

## Conflict of Interest

The authors declare that the research was conducted in the absence of any commercial or financial relationships that could be construed as a potential conflict of interest.

## Publisher’s Note

All claims expressed in this article are solely those of the authors and do not necessarily represent those of their affiliated organizations, or those of the publisher, the editors and the reviewers. Any product that may be evaluated in this article, or claim that may be made by its manufacturer, is not guaranteed or endorsed by the publisher.
